# Label-Free and Ultra-Sensitive Detection of Dexamethasone Using a FRET Aptasensor Utilizing Cationic Conjugated Polymers

**DOI:** 10.3390/bios14080364

**Published:** 2024-07-26

**Authors:** Yizhang Xue, Hangbing Liu, Ye Zhang, Weijun Yang, Huixin Li, Yuxuan Gong, Yubai Zhang, Bo Li, Chang Liu, Yi Li

**Affiliations:** 1 Sport Coaching College, Beijing Sport University, Beijing 100084, China; 2021010099@bsu.edu.cn (Y.X.); 2021010103@bsu.edu.cn (Y.Z.); yangweijun@bsu.edu.cn (W.Y.);; 2 Beijing Institute of Pharmacology and Toxicology, Beijing 100850, China; liuhb133@163.com; 3 School of Pharmaceutical Engineering, Shenyang Pharmaceutical University, Shenyang 110016, China; 4 School of Sport Science, Beijing Sport University, Beijing 100084, China; 2021011319@bsu.edu.cn (H.L.);; 5 Division of Sport Science and Physical Education, Tsinghua University, Beijing 100084, China

**Keywords:** dexamethasone, label-free detection, Förster Resonance Energy Transfer (FRET), aptasensor, anti-doping

## Abstract

Dexamethasone (Dex) is a widely used glucocorticoid in medical practice, with applications ranging from allergies and inflammation to cerebral edema and shock. Despite its therapeutic benefits, Dex is classified as a prohibited substance for athletes due to its potential performance-enhancing effects. Consequently, there is a critical need for a convenient and rapid detection platform to enable prompt and accurate testing of this drug. In this study, we propose a label-free Förster Resonance Energy Transfer (FRET) aptasensor platform for Dex detection utilizing conjugated polymers (CPs), cationic conjugated polymers (CCPs), and gene finder probes (GFs). The system operates by exploiting the electrostatic interactions between positively charged CCPs and negatively charged DNA, facilitating sensitive and specific Dex detection. The label-free FRET aptasensor platform demonstrated robust performance in detecting Dex, exhibiting high selectivity and sensitivity. The system effectively distinguished Dex from interfering molecules and achieved stable detection across a range of concentrations in a commonly used sports drink matrix. Overall, the label-free FRET Dex detection system offers a simple, cost-effective, and highly sensitive approach for detecting Dex in diverse sample matrices. Its simplicity and effectiveness make it a promising tool for anti-doping efforts and other applications requiring rapid and accurate Dex detection.

## 1. Introduction

Dexamethasone (Dex) is a potent glucocorticoid widely utilized in medical practice [1]. Its applications encompass a wide range of medical conditions, including allergies, inflammation, cerebral edema, acute exacerbations of multiple sclerosis, and shock [2,3,4,5,6]. Patients suffering from ailments such as contact dermatitis, atopic dermatitis, asthma, and drug allergies can benefit from the therapeutic effects of Dex [7,8,9,10]. Furthermore, in the realm of endocrinology, Dex serves as a valuable diagnostic tool for Cushing’s syndrome [11]. Additionally, it is utilized in the management of chemotherapy-induced nausea and vomiting, as well as in the prevention and treatment of altitude sickness [12,13]. Moreover, it is employed in the treatment of spinal cord compression resulting from tumor metastases [14]. Notably, even in severe cases of Coronavirus Disease 2019 (COVID-19) necessitating supplemental oxygen or ventilatory support, Dex demonstrates favorable clinical outcomes [15]. In essence, Dex exhibits broad therapeutic efficacy, exerting its influence on patient health through diverse biological mechanisms [1].

Regrettably, Dex is classified as a prohibited substance for athletes by multiple sports governing bodies, including the World Anti-Doping Agency (WADA) and the International Convention against Doping in Sport formulated by the United Nations Educational, Scientific and Cultural Organization (UNESCO) [16]. Managing its use poses significant complexity compared to other substances prohibited both in and out of competition settings, such as anabolic androgenic steroids, erythropoietin, and growth hormone, which are absolutely forbidden. Specifically, Dex is prohibited from oral administration during competitions, encompassing various intra-oral mucosal routes such as buccal, gingival, and sublingual administration. Furthermore, administration via injection or rectal routes is strictly prohibited during competitions. However, alternative administration methods during competitions remain unrestricted, and there are no restrictions on out-of-competition usage [17].

Specifically, unlike doping drugs that are absolutely prohibited for athletes, Dex is more like a commonly used medical drug that requires timely and effective control. This means it should be used only where permitted, with accidental ingestion and misuse strictly prohibited. Furthermore, since it is nearly impossible to determine the chemical composition of tablets, capsules, or ointments by appearance alone, the risk of accidentally ingesting Dex is significantly higher compared to other doping drugs.

As a result, athletes often come into contact with Dex in their living environments, leading to frequent positive test results. Nonetheless, the intent behind its usage, whether intentional or inadvertent, remains uncertain, underscoring the critical need for a convenient and rapid detection platform to enable prompt and accurate testing of this drug. Such measures are crucial to prevent athletes from unintentionally using or being exposed to Dex, as demonstrated by scenarios where an athlete applies Dex topically and subsequently handles food or drink items consumed by another athlete during competition, without their knowledge.

Currently, the method endorsed by WADA for Dex detection is high-performance liquid chromatography-mass spectrometry (HPLC-MS) [18]. However, this method encounters challenges associated with instrumentation, including prolonged analysis times, high equipment costs, and stringent testing environment requirements. These factors limit its widespread application and practical utility under constrained experimental conditions [19]. Therefore, from the athletes’ perspective, there is an urgent need to develop ultra-sensitive techniques for detecting Dex in food or drink items.

Meanwhile, it is important to note that the purpose of this study is not to detect Dex in athletes’ urine to prove doping violations, as is conducted by organizations like WADA and UNESCO. Those studies have well-established and highly accurate detection methods that can be used as evidence in international courts. Instead, the application scenario of this study is to provide athletes with a fast and portable detection platform. This platform can quickly identify whether drinks, food, nutritional supplements, or cosmetics around the athletes are contaminated with Dex. If contamination is detected, the athletes can immediately stop using these items to prevent Dex from entering their bodies and causing doping violations.

Consequently, various straightforward and rapid analytical methods for detecting Dex, such as enzyme-linked immunosorbent assay (ELISA) [20], electrochemistry [21], and chemiluminescence [22] have been introduced. Although these methods are not as precise and accurate as HPLC-MS—which, for example, has a linear range as low as 1.27 × 10^−6^ μM to 1.53 × 10^−4^ μM—they still hold significant importance and serve complementary roles to varying degrees [23]. 

The ELISA method involves coupling Dex with hemocyanin, bovine serum albumin, and horseradish peroxidase. Detection is performed using both one-step and two-step double antibody ELISA methods, as well as radioimmunoassay, with a detection range from 2.55 × 10^−5^ μM to 1.27 × 10^−1^ μM [24]. Electrochemical methods, through experimental and theoretical studies of the electrochemical behavior on graphene-modified glassy carbon electrodes, utilize voltammetry techniques to detect Dex. This approach exhibits two linear dynamic ranges: 0.1–50 μM and 50–5000 μM [21]. The chemiluminescence method utilizes the light produced by the reaction of KMnO_4_ and L-cysteine-modified CdS quantum dots in an acidic medium as a simple and sensitive chemiluminescence system for the determination of Dex. This method offers a linear range from 1.02 × 10^−2^ μM to 6.372 × 10^−1^ μM [22].

These methods have significantly advanced the detection of Dex, each enhancing the accuracy and speed of its detection in their own unique ways. However, it is noteworthy that these technologies often entail labeling procedures, which not only escalate analytical costs but may also compromise the specificity and affinity of Dex-related probes. Hence, there persists a pressing need to devise a simple, cost-effective, and ultra-sensitive Dex detection method capable of circumventing labeling steps.

Conjugated polymers (CPs) represent a class of organic semiconductor materials known for their extensive delocalized π-conjugated backbones. These backbones possess desirable light-harvesting and light-amplifying properties, making CPs widely utilized in the biological field. To improve their applicability in biological settings, cationic conjugated polymers (CCPs) are synthesized by introducing positive charges onto the side chains of CPs. This cationic property enables CCPs to dissolve in biological solutions and effectively interact with deoxyribonucleic acid (DNA) through electrostatic interactions [25]. 

Förster Resonance Energy Transfer (FRET) is a non-radiative process that takes place between a donor and an acceptor molecule [26]. Typically, the emission spectrum of the donor overlaps with the excitation spectrum of the acceptor [27]. Through long-range dipole–dipole interactions, the excited donor transfers energy to the acceptor. The acceptor absorbs energy at the wavelength emitted by the donor but does not necessarily fluoresce itself to emit energy [28]. FRET typically occurs within distances ranging from 10 to 100 angstrom (Å), which is comparable to the size of most biological macromolecules [29]. As a result, utilizing CCPs in FRET aptasensors can sensitively detect various biomarkers, such as DNA methylation [30] and disease-related proteins [31]. Furthermore, the label-free property of a genefinder-based aptasensor can reduce analysis costs, presenting a potentially cost-effective approach for simple and highly sensitive Dex detection.

Poly (9,9-bis(6′-N,N,N-trimethylammonium)hexyl)fluorene-co-alt-1,4-phenylene) bromide (PFP) functions as an energy donor in this context [32]. PFP is a water-soluble CCP. In comparison to its small molecule counterparts, the CCP backbone exhibits a delocalized electronic structure and encompasses numerous absorption units. Excitation energy along the CCP backbone can be transferred to the acceptor molecule through either electron transfer or FRET mechanisms [33]. This transfer may lead to the efficient quenching of CCP fluorescence or the amplification of the acceptor fluorescence signal [34]. Moreover, CCP has an affinity for negatively charged nucleic acids due to electrostatic interactions [35]. As a consequence, efficient FRET can occur from CCP to fluorophore-labeled oligonucleotide probes.

**Figure 1:**
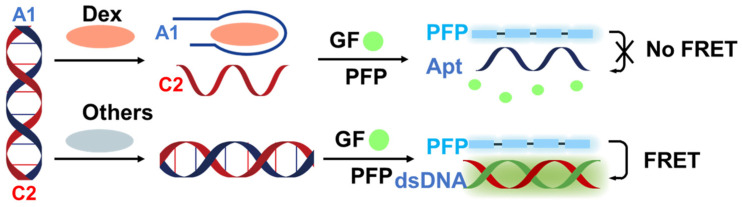
Schematic representation of the assay designed for the label-free detection of dexamethasone utilizing cationic conjugated polymers via the FRET strategy. A1 represents the dexamethasone aptamer, C2 represents the complementary DNA of the dexamethasone aptamer, Dex represents dexamethasone, GF represents gene finder, PFP represents Poly(9,9-bis(6′-N,N,N-trimethylammonium)hexyl)fluorene-co-alt-1,4-phenylene) bromide, dsDNA represents double-stranded DNA, and FRET represents Förster Resonance Energy Transfer.

Here, we propose a label-free FRET aptasensor platform for Dex detection utilizing PFP, aptamers, and gene finder probes (GFs). As illustrated in Figure 1, prior to the detection process, the Dex aptamer (A1) and complementary DNA (C2) were designed and formed into a double-stranded DNA complex [36]. In the absence of the target molecule, the aptamer still forms a stable duplex with C2. GFs can then intercalate into the double-stranded DNA, leading to over 100-fold fluorescence enhancement compared to the free state. The electrostatic interaction between the positively charged PFP and the negatively charged backbone of the double-stranded DNA reduces the distance between PFP and GF, resulting in significant quenching of PFP fluorescence due to FRET from PFP to GF, thereby enhancing the fluorescence intensity of GF.

Upon the presence of Dex, the double-stranded DNA breaks down, causing GF to dissociate from the DNA. Subsequently, A1 forms a more specific and stable complex with Dex than with C2. Consequently, the free state of GF ultimately disrupts the FRET system. Therefore, by evaluating the extent of FRET quenching, ultrasensitive quantitative detection of Dex can be achieved.

## 2. Materials and Methods

### 2.1. Materials and Instrumentation

Dexamethasone (Catalog No. D1756, Sigma-Aldrich, St. Louis, MO, USA) possesses a theoretical solubility of approximately 267 μM, owing to its limited water solubility of roughly 100 mg/L and molar mass of about 374.48 g/mol. Accordingly, we prepared 25 mL stock solutions of Dex by dissolving it in double-distilled H_2_O (ddH_2_O, produced by Milli-Q^®^ EQ 7000 Ultrapure Water Purification System, Merck KGaA, Darmstadt, Germany) with 4% (*v*/*v*) dimethyl sulfoxide (DMSO, Catalog No. D8370, Solarbio, Beijing, China) to achieve a concentration of 2500 μM. Subsequently, we conducted serial dilutions to obtain concentrations of 2500 μM, 500 μM, 50 μM, 2.5 μM, and 0.25 μM with 25 mL of each group.

The pre-solution containing mixed double-stranded DNA consisted of 100 µM Dex aptamer (A1, 5′-ACACGACGAGGGACGAGGAGTACTTGCCAACGATAACGTCGTGGATCTGTCTGTGCCC-3′, Thermo Fisher Scientific Inc., Waltham, MA, USA) and 100 µM complementary DNA (C2, 5′-GGGCACAGACAGATCCAACGACGTTATCGTTGGCAAGTACTCCTCGTCCCTCGTCGTGT-3′, Thermo Fisher Scientific Inc., Waltham, MA, USA) in 10 mL double-distilled H_2_O mixtures. To facilitate the formation of mixed double-stranded DNA, the mixed solution was heated to over 90 °C and then allowed to naturally cool to room temperature (20 °C). Testosterone, progesterone, 17β-estradiol, estriol, and salidroside were prepared as stock solutions by dissolving them separately in ddH_2_O to achieve a concentration of 250 μM with 25 mL of each group.

Genefinder (Catalog No. D039, Bridgen, Beijing, China) was diluted from a 10,000-fold concentration when purchased to a 10-fold dilution in a 10 mL stock solution, using ddH_2_O serving as the dilution medium. PFP is a compound synthesized and previously published by our research group [37], with its synthesis route derived from authoritative literature [38]. PFP was dissolved in 4% (*v*/*v*) dimethyl sulfoxide (DMSO, Catalog No. D8370, Solarbio, Beijing, China)-ddH_2_O mixtures, as recommended by literature, and is prepared for use at a concentration of 40 µM of 5 mL stock. 

The UV-Vis absorption spectra and fluorescence spectra were recorded using a Spark^®^ Multimode Microplate Reader (Tecan Trading AG, Männedorf, Switzerland).

### 2.2. Detection Assay

The pre-solution containing the Dex aptamer (A1) and its complementary sequence (C2) was prepared by mixing with various concentrations of dexamethasone (2500 μM, 500 μM, 50 μM, 2.5 μM, and 0.25 μM), or a negative control consisting of ddH_2_O only with a 6:2 ratio, which means 600 μL of the pre-solution and 200 μL of Dex, for instance. These mixtures were then co-incubated at room temperature (20 °C) for 2 h. Subsequently, 100 μL PFP (40 µM) and 100 μL GF (10×) were added to the solutions to achieve final concentrations of PFP at 4 µM and GF at 1× for testing the FRET. After dilution, the final concentrations of Dex in the working solution were 500 μM, 100 μM, 10 μM, 0.5 μM, and 0.05 μM.

The fluorescence intensity was measured to evaluate the extent of quenching in the FRET system. Excitation was performed at a wavelength of 380 nm, and the emission spectrum was recorded within the range of 400 to 650 nm. Normalization was performed by identifying the highest fluorescence signal between 424 and 538 nm across various assays.

### 2.3. Selective Assay

In a similar manner, the pre-solution containing the mixture of A1 and C2 was prepared. Test agents or other potential interfering substances, including testosterone, progesterone, 17β-estradiol, estriol, and salidroside, were added to the pre-solution at a ratio of 2:6, respectively, which means 200 μL of drugs and 600 μL of the pre-solution, for example, based on experimental grouping (experimental and control groups). These mixtures were then co-incubated at room temperature (20 °C) for 2 h. Subsequently, 100 μL PFP (40 µM) and 100 μL GF (10×) were added to the solutions to achieve final concentrations of PFP at 4 µM and GF at 1× for testing the FRET.

Fluorescence intensity was measured to assess the degree of quenching in the FRET system. Excitation was performed at a wavelength of 380 nm, and the emission spectrum was recorded within the range of 400 to 650 nm. Normalization was conducted by identifying the highest fluorescence signal between 424 and 538 nm across various assays.

### 2.4. Selective Assay

All measurements were conducted in at least three independent experiments, and the results are expressed as mean ± standard deviation (mean ± SD). Statistical analysis was performed using the Student’s *t*-test (*n* ≥ 3) with the utilization of Statistical Package for the Social Sciences (SPSS) version 22.0. Significance levels were set at * *p* < 0.05 and ** *p* < 0.01 [39].

## 3. Results

### 3.1. Characterization of FRET Phenomenon in PFP/GF System

**Figure 2:**
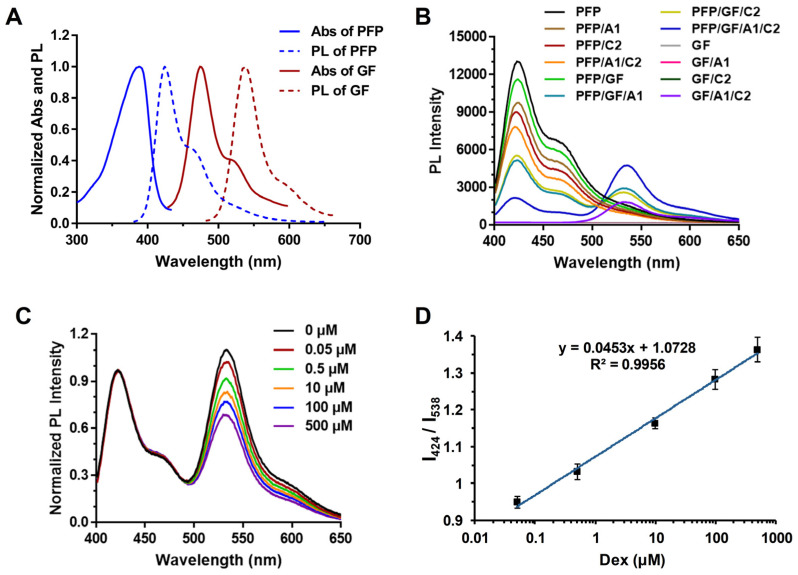
Schematic representation of the assay devised for the label-free detection of dexamethasone (Dex) utilizing the cationic conjugated polymers through the Förster Resonance Energy Transfer (FRET) strategy; (**A**) Absorption spectra (Abs) and Photoluminescence spectra (PL) of PFP and gene finder (GF); (**B**) The FRET phenomenon occurring between Poly (9,9-bis(6′-N,N,N-trimethylammonium)hexyl)fluorene-co-alt-1,4-phenylene) bromide (PFP) and GF in the presence of DNA (A1, C2, or A1 + C2); (**C**) The normalized fluorescence outcomes of the FRET system at varying concentrations of Dex; (**D**) The corresponding linear correlation between the PL intensity at 424 nm/PL intensity at 538 nm (I_424_/I_538_) ratio and Dex concentration. Each assay underwent a minimum of three repetitions, and the results are presented as mean values with standard deviation (±SD).

As previously mentioned, the occurrence of the FRET phenomenon relies on the overlap between the photoluminescence spectrum (PL) of the donor molecule and the absorption spectrum (Abs) of the acceptor molecule. Therefore, this study initially investigated this phenomenon. As depicted in Figure 2A, the fluorescence spectrum of PFP and the absorption spectrum of GF were evaluated, revealing a significant overlap around 460 nm. This observation fulfills the criteria for FRET.

As illustrated in Figure 2B, the aim was to simulate the final experimental system to determine whether the FRET phenomenon would occur in a system containing PFP, GF, A1, and C2 simultaneously. Multiple sets of experiments were designed, including PFP, PFP/A1, PFP/C2, and PFP/A1/C2. It was observed that adding A1 or C2 alone, or even both A1 and C2 simultaneously, did not induce noticeable changes in the overall fluorescence spectrum. Similarly, experiments involving GF, GF/A1, GF/C2, and GF/A1/C2 demonstrated that the addition of A1 or C2 alone did not affect GF fluorescence, but the simultaneous addition of both A1 and C2 resulted in GF binding and embedding into the DNA double strand, leading to a distinct fluorescent signal. However, this signal was notably not due to FRET.

Likewise, adding A1 or C2 alone to the PFP or GF systems (PFP/A1, PFP/C2, GF/A1, and GF/C2) did not produce observable FRET phenomena. Ultimately, the FRET phenomenon was only observed in the mixed system containing PFP/GF with the presence of A1 and/or C2 (PFP/GF/A1, PFP/GF/C2, and PFP/GF/A1/C2). Specifically, under excitation at approximately 424 nm, a significant decrease in fluorescence intensity associated with PFP was observed, accompanied by the emergence of a fluorescence signal around 538 nm. Hence, the observed FRET phenomenon confirms that, owing to the overlap in band gaps between PFP and GF, PFP can bind to DNA and subsequently serve as an energy donor, transferring energy to GF within the double-stranded DNA (dsDNA) structure.

Based on the successful establishment of the aforementioned system, when A1 and C2 complementarily match to form a DNA double helix, GF can effectively insert DNA and produce a specific intensity of fluorescence. Due to the abundance of positive charge in PFP, it can attract the dsDNA to a relatively close distance. Simultaneously, the fluorescence spectrum of the donor molecule PFP overlaps with the absorption spectrum of the receptor molecule GF, resulting in the occurrence of FRET. This phenomenon diminishes the fluorescence intensity of PFP while significantly enhancing the fluorescent signal of GF.

Therefore, based on the principles mentioned above, when Dex is introduced into the system, it promptly forms specific recognition with A1. A1 bound to Dex fails to form a DNA double helix with C2, thereby attenuating the FRET phenomenon. Consequently, the fluorescence of GF around 538 nm diminishes.

### 3.2. Evaluation of the Detection Method for Dex

To investigate whether the addition of Dex correlates linearly with the quenching level of the FRET system, we introduced varying concentrations of Dex (2500 μM, 500 μM, 50 μM, 2.5 μM, 0.25 μM, and ddH_2_O) into a pre-solution containing A1 and C2 according to the aforementioned methods. Subsequently, we added PFP (40 μM) and GF (10×) to the solutions to achieve final concentrations of Dex (500 μM, 100 μM, 10 μM, 0.5 μM, 0.05 μM, and ddH_2_O), along with A1 and C2. This allowed sufficient time for A1 and Dex to fully interact and disrupt the DNA double helix formed by A1 and C2.

As depicted in Figure 2C, upon Dex addition, the fluorescence of GF around 538 nm progressively decreases with increasing Dex concentration. The fluorescence intensity of PFP at 424 nm remains relatively unchanged, serving as a stable internal control. Specifically, in this detection system, when PFP, GF, A1, and C2 are all present, a DNA double helix is formed between A1 and C2, GF is anchored in the DNA double helix, and PFP closes the distance between GF and DNA through electrostatic interaction, resulting in FRET, which increases fluorescence at 538 nm. When Dex is added, it interacts with A1 and binds to it. This binding competitively prevents the interaction between A1 and C2, so a DNA double helix cannot be formed. Consequently, GF cannot be inserted into the DNA double helix, significantly weakening the FRET of the entire system. As we can see from Figure 2C, as the concentration of Dex increases, the fluorescence intensity at 538 nm decreases significantly. Consequently, the ratio of intensities (I_424_/I_538_) at 424 nm and 538 nm is utilized for quantification to form Figure 2D. As shown in Figure 2D, a standard curve is plotted with Dex concentration as the x-axis and I_424_/I_538_ as the y-axis. 

The findings demonstrate that the detection method displays a remarkable linear response to digital linearity within the range of 0.05 to 500 μM, with the lowest limit of detection (LOD) determined to be 0.024 μM. This FRET strategy demonstrates a superior linear range and lower LOD value. It offers relatively high accuracy, while also circumventing the necessity for extensive instrumentation, rendering it cost-effective. Additionally, it exhibits significant advancements in application potential, accompanied by distinct advantages.

### 3.3. Selectivity Confirmation in Dex Detection

**Figure 3:**
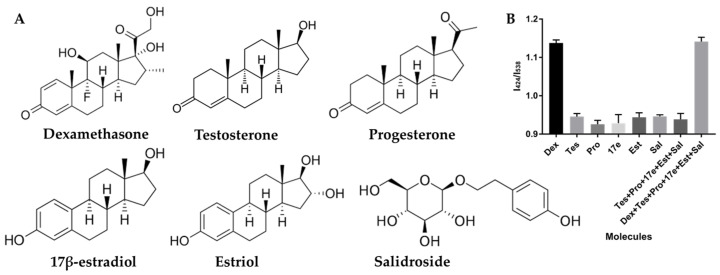
Schematic representation of selectivity confirmation in the detection of dexamethasone (Dex); (**A**) Molecular structures of Dex and several common interfering molecules; (**B**) Ratio of fluorescence intensities (I_424_/I_538_) observed with Dex and the interfering molecules. Concentrations of Dex and interfering molecules are maintained at 20 μM. Tes represents the Testosterone, Pro represents the progesterone, 17e represents the 17β-estradiol, Est represents the estriol, and Sal represents the salidroside.

Based on literature reports, the A1 used in this study for Dex exhibits notable selectivity. To further confirm its specificity and ability to avoid interference from other molecules within this system, several common interfering molecules were selected and introduced into the system to evaluate its selectivity towards these compounds. These molecules include testosterone (Tes), progesterone (Pro), 17β-estradiol (17e), estriol (Est), and salidroside (Sal), as depicted in Figure 3A. The test outcomes are presented in Figure 3B. Remarkably, at a consistent concentration of 20 μM, the I_424_/I_538_ ratios of the Dex group were markedly higher compared to other groups, indicating excellent selectivity. Furthermore, we introduced the aforementioned molecules as interfering agents and incubated them with Dex separately, thereby creating systems containing five or all molecules. Encouragingly, the presence of these similar molecules did not compromise the accuracy of the results. Collectively, these findings unequivocally establish the high selectivity of this label-free FRET-based method for Dex determination.

### 3.4. Assessment of Method Accuracy in Realistic Environments

To further assess the accuracy of this system in practical settings, a recovery experiment was conducted. Dex was added to a commercially available energy drink to simulate common environments encountered by athletes, which are particularly vulnerable to doping contamination. The method was applied to detect Dex at three concentrations: low (0.02 μM), medium (2.0 μM), and high (200 μM). As illustrated in Table 1, the results indicate that the method effectively detects Dex in the liquid environments typical of athletes’ real-life scenarios. It demonstrates a favorable recovery rate ranging from 99.6 to 104, with a relative standard deviation (RSD) of less than 3%.

In summary, the nucleic acid aptamer based on PEP and GF offers several advantages, including low cost, selectivity, stability, and high recovery rate. These characteristics make it highly suitable for large-scale screening applications.

## 4. Discussion

In this investigation, a label-free FRET aptamer sensor, employing PFP and GF, was developed for ultrasensitive Dex detection via a straightforward process. Utilizing the nucleic acid aptamer A1, as reported in the literature, which effectively binds to Dex, and designing complementary sequence C2, when A1 and C2 form a complementary double-stranded DNA, GF can be intercalated into the double-stranded DNA, resulting in a detectable fluorescent signal. PFP, due to its positive charge, facilitates DNA attraction, thus shortening the distance between DNA and PFP. Additionally, the emission spectrum of PFP precisely overlaps with the absorption spectrum of GF, enabling effective FRET occurrence, and consequently enhancing the fluorescence intensity of GF. Notably, a robust fluorescence signal was observed around 538 nm.

Upon introduction of Dex into the system, it binds to its corresponding nucleic acid aptamer A1 to form a more stable complex, preventing A1 from pairing with C2 and leading to the dissociation of GF from DNA, thereby quenching the FRET phenomenon. Consequently, diminished fluorescence intensity ensues.

At the same time, we found that the change in fluorescence intensity caused by FRET and the amount of Dex exhibited a linear relationship, which can be used for quantitative analysis. The experimental results demonstrated that Dex had a good linear range from 0.05 μM to 500 μM. Although this method is not as accurate and precise as HPLC-MS, which is recognized by WADA and UNESCO as court evidence, it has a comparable order of magnitude of sensitivity and accuracy to the ELISA, electrochemistry, and chemiluminescence methods mentioned above. Like these methods, it provides an instant measurement that allows athletes to quickly and timely detect the presence of doping substances in their food, beverages, and nutritional supplements. It offers rapid test results without relying on large instruments and is cost-effective.

Subsequently, various interfering molecules were introduced into the FRET detection platform. Remarkably, the system exhibited high selectivity exclusively towards Dex, with no fluorescence quenching observed with other molecules. Furthermore, the addition of these interfering molecules to the Dex solution did not compromise the system’s selectivity and sensitivity, indicating its ability to resist interference from common interfering molecules and achieve stable Dex detection.

To further validate the system’s applicability in the sports industry, particularly in anti-doping endeavors, the study employed a commonly used sports drink as a solvent, spiked with Dex, and subjected it to detection. Notably, stable and sensitive detection was achieved across low, medium, and high concentrations, with a commendable recovery rate of 99.6 to 104 and a relative standard deviation of less than 3, demonstrating the method’s effectiveness in Dex detection.

It is worth noting that the WADA-approved detection method for dexamethasone is typically a urine test. Frequent blood draws from athletes can be relatively detrimental to their welfare. Although there may be significant protein interference in the system, this interference is usually removed by pre-treatment methods. Consequently, this study does not consider potential protein or serum interference. As our research progresses, we aim to continue supplementing related work, thereby better supporting global anti-doping efforts and protecting the welfare and well-being of athletes.

Consequently, the label-free FRET Dex detection system presented herein eliminates the need for specialized labeling processes such as modification, coating, fixation, or separation, offering simplicity and cost-effectiveness. Thus, the PEP-based label-free FRET nucleic acid aptamer sensor delineated in this study showcases a simple procedure, affordability, high selectivity, exceptional sensitivity, and specificity, facilitating rapid and widespread Dex detection across diverse sample matrices.

## 5. Conclusions

In conclusion, this study presents a label-free FRET aptamer sensor utilizing PFP and GF for ultrasensitive detection of Dex. The system demonstrated remarkable selectivity towards Dex, with negligible interference from other molecules. Label-free FRET aptamer sensors hold immense promise as a versatile platform for highly specific and sensitive biodetection due to their inherent stability, high selectivity, solvent compatibility, simplicity, low background noise, and even cost-effectiveness. Furthermore, advancements in specificity and integration of label-free FRET aptamer sensors hold tremendous potential for portable and point-of-care diagnostic applications. Although preliminary, the current work provides practical evidence for the design and application of label-free FRET aptamer sensors as a powerful and versatile platform for bioanalytical applications.

## Figures and Tables

**Table 1:** Recovery Rates and RSD Results of Dex in Authentic Samples Utilizing FRET Method based on PEP and GF. RSD represents the relative standard deviation.

Sample	Spiked Amount (μM)	Measured Value (μM)	Recovery (%)	RSD (%)
1	0.0200	0.0208	104	2.89
2	2.00	2.04	102	2.70
3	200	199.2	99.6	0.33

## Data Availability

Data are contained within the article.
